# Frequency of GAA-*FGF14* Ataxia in a Large Cohort of Brazilian Patients With Unsolved Adult-Onset Cerebellar Ataxia

**DOI:** 10.1212/NXG.0000000000200094

**Published:** 2023-08-28

**Authors:** Luiz Eduardo Novis, Rodrigo S. Frezatti, David Pellerin, Pedro J. Tomaselli, Shahryar Alavi, Marcus Vinícius Della Coleta, Mariana Spitz, Marie-Josée Dicaire, Pablo Iruzubieta, José Luiz Pedroso, Orlando Barsottini, Andrea Cortese, Matt C. Danzi, Marcondes C. França, Bernard Brais, Stephan Zuchner, Henry Houlden, Salmo Raskin, Wilson Marques, Helio A. Teive

**Affiliations:** From the Pós-graduação Em Medicina Interna e Ciências da Saúde (L.E.N., H.A.T.), Hospital de Clínicas da Universidade Federal do Paraná, Curitiba, Brazil; Department of Neuromuscular Diseases (L.E.N., D.P., S.A., P.I., A.C., H.H.), UCL Queen Square Institute of Neurology and the National Hospital for Neurology and Neurosurgery, London, UK; Department of Neurology (R.S.F., P.J.T., W.M.), School of Medicine at Ribeirão Preto, University of São Paulo, Ribeirão Preto, Brazil; Departments of Neurology and Neurosurgery (D.P., M.-J.D., B.B.), Montreal Neurological Hospital and Institute, McGill University, Canada; Departamento de Neurologia (M.V.D.C.), Universidade do Estado do Amazonas, Manaus; Departamento de Especialidades Médicas (M.S.), Serviço de Neurologia, Universidade Estadual do Rio de Janeiro, Brazil; Department of Neurology (P.I.), Donostia University Hospital; Neuroscience Area (P.I.), Biodonostia Health Research Institute, San Sebastian; Network Center for Biomedical Research in Neurodegenerative Diseases (CIBERNED) (P.I.), Spain; Department of Neurology (J.L.P., O.B.), Ataxia Unit, Universidade Federal de São Paulo, SP, Brazil; Department of Brain and Behavioral Sciences (A.C.), University of Pavia, Italy; Dr. John T. Macdonald Foundation Department of Human Genetics and John P. Hussman Institute for Human Genomics (M.C.D., S.Z.), University of Miami Miller School of Medicine; Department of Neurology (M.C.F.), School of Medical Sciences-University of Campinas (UNICAMP), São Paulo, Brazil; Department of Human Genetics (B.B.), McGill University, Montreal, Canada; and Laboratório Genetika (S.R.), Curitiba, PR, Brazil.

## Abstract

**Objectives:**

Intronic *FGF14* GAA repeat expansions have recently been found to be a common cause of hereditary ataxia (GAA-*FGF14* ataxia; SCA27B). The global epidemiology and regional prevalence of this newly reported disorder remain to be established. In this study, we investigated the frequency of GAA-*FGF14* ataxia in a large cohort of Brazilian patients with unsolved adult-onset ataxia.

**Methods:**

We recruited 93 index patients with genetically unsolved adult-onset ataxia despite extensive genetic investigation and genotyped the *FGF14* repeat locus. Patients were recruited across 4 different regions of Brazil.

**Results:**

Of the 93 index patients, 8 (9%) carried an *FGF14* (GAA)_≥250_ expansion. The expansion was also identified in 1 affected relative. Seven patients were of European descent, 1 was of African descent, and 1was of admixed American ancestry. One patient carrying a (GAA)_376_ expansion developed ataxia at age 28 years, confirming that GAA-*FGF14* ataxia can occur before the age of 30 years. One patient displayed episodic symptoms, while none had downbeat nystagmus. Cerebellar atrophy was observed on brain MRI in 7 of 8 patients (87%).

**Discussion:**

Our results suggest that GAA-*FGF14* ataxia is a common cause of adult-onset ataxia in the Brazilian population, although larger studies are needed to fully define its epidemiology.

## Introduction

Dominantly inherited GAA repeat expansions in intron 1 of the fibroblast growth factor 14 (*FGF14*) gene have recently been shown to be a common cause of adult-onset ataxia (GAA-*FGF14* ataxia; spinocerebellar ataxia 27B) in European and South Asian populations.^1,2^ Patients with GAA-*FGF14* ataxia displayed a slowly progressive cerebellar syndrome with frequent episodic symptoms and downbeat nystagmus.^1,2^

In Brazil, SCA3 is the most prevalent form of hereditary ataxia, followed by SCA2 and SCA10.^3,4^ The high prevalence of SCA3 is largely due to the Portuguese-Azorean founder effect, while SCA2 and SCA10 originate from the native American populations.^3-5^ The ethnic diversity of the Brazilian population provides a unique opportunity to study the distribution and prevalence of GAA-*FGF14* ataxia. In this study, we investigated the frequency and phenotypic profile of GAA-*FGF14* ataxia in a large cohort of Brazilian patients with unsolved adult-onset ataxia.

## Methods

Ninety-three index patients were recruited in Curitiba (Hospital de Clínicas da Universidade Federal do Paraná), Rio de Janeiro (Hospital Universitário Pedro Ernesto), Amazonas (Universidade do Estado do Amazonas), and São Paulo (Hospital das Clínicas da Faculdade de Medicina de Ribeirão Preto) in Brazil. Patients presenting with unsolved neurodegenerative ataxia with onset after the age of 20 years were enrolled. Patients had previously undergone comprehensive investigation to exclude acquired and genetic causes, which included whole-exome sequencing (42/93; the list of analyzed genes was based on the study conducted by Sun et al.^6^) and screening for SCA1, SCA2, SCA3, SCA6, SCA7, SCA10, *RFC1*-related ataxia, and Friedreich ataxia. The *FGF14* repeat locus was genotyped as previously described.^7^ Expansions of at least 250 GAA repeat units were considered pathogenic.^1,2^

### Standard Protocol Approvals, Registrations, and Patient Consents

We obtained written informed consent from all participants in the study, and the institutional review board of the Hospital de Clínicas da Universidade Federal do Paraná and Hospital das Clínicas da Faculdade de Medicina de Ribeirão Preto approved this study.

### Data Availability

Individual anonymized data may be shared at the request of any qualified investigator on reasonable request.

## Results

We identified 8 index patients (8/93; 9%) who carried an *FGF14* (GAA)_≥250_ expansion (Figure). The expansion was also identified in the affected son (patient 1.2) of an affected mother (patient 1.1). In this family, the transmission of an expanded allele resulted in expansion in the female germline (expansion from 253 to 268 repeats), consistent with previous reports.^1,2,7^ We also identified a patient carrying biallelic GAA repeat expansions (363 and 448 repeats). Ancestry analysis of short-read sequencing data^8^ showed that 1 patient was of admixed American ancestry and 2 patients were of European ancestry. One patient self-reported being of African descent, while the remaining patients self-reported being of European descent.

**Figure:**
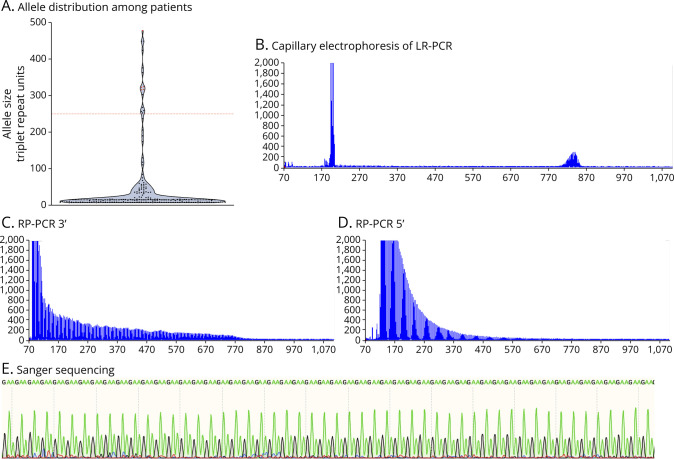
Allele Distribution and Genetic Analysis of the *FGF14* GAA Repeat Locus (A) Allele distribution of the *FGF14* repeat locus in 94 Brazilian patients with unsolved adult-onset ataxia. The repeat length was measured by capillary electrophoresis of fluorescent long-range PCR amplification products. The allele sizes are expressed in numbers of triplet repeat units. Expanded alleles consisting of non-GAA repeats are represented by red triangles. The dashed red line indicates the pathogenic threshold of at least 250 GAA repeat units. The bold dashed black line within the violin plot shows the median allele size. (B) Fragment length analysis results of patient 1.1 carrying an expanded allele of 253 GAA repeat units. (C and D) Results of repeat-primed PCR targeting the (C) 3′-end and (D) 5′-end of the *FGF14* repeat locus in patient 1.1. (E) Excerpt of Sanger sequencing chromatogram showing the GAA motif of the expanded allele of patient 1.1.

Table summarizes the main features of the 9 patients with GAA-*FGF14* ataxia. The median age at onset was 59 years (range, 28–67 years). We identified 1 patient who had an age at disease onset of 28 years, confirming that GAA-*FGF14* ataxia can begin before the age of 30 years. All patients displayed a slowly progressive cerebellar syndrome, although none had dysarthria. A single patient experienced episodic symptoms, which were triggered by alcohol intake and stress, while 56% of patients (5/9) reported diplopia and 44% (4/9) reported vertigo and/or dizziness. Horizontal gaze-evoked nystagmus was observed in 78% of cases (7/9), consistent with previous reports showing that cerebellar oculomotor signs are common in GAA-*FGF14* ataxia.^1,9^ However, none of the 9 patients exhibited downbeat nystagmus. The patient carrying biallelic GAA repeat expansions had a relatively rapid disease progression (spinocerebellar degeneration functional score [SDFS] disability score^10^ of 3 after 3-year disease duration, indicating moderate gait impairment). In addition, extrapyramidal features were observed in 2 patients.

**Table:**
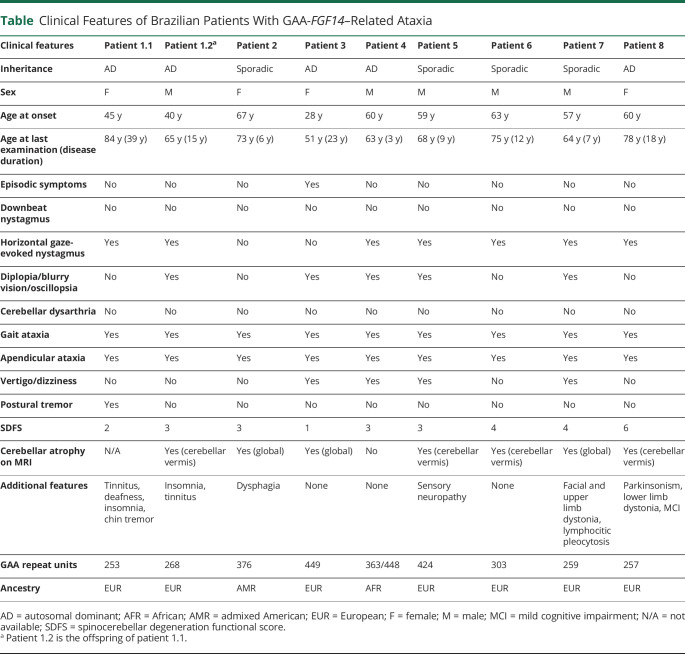
Clinical Features of Brazilian Patients With GAA-*FGF14*–Related Ataxia

Overall, the median disease duration was 12 years (range, 3–39 years). Two patients required unilateral walking aid after 7 and 12 years of disease duration, respectively. One patient became wheelchair bound after long-standing disease (15 years), in keeping with previous reports showing that wheelchair dependence is uncommon in GAA*-FGF14* ataxia.^9^ All other patients were still ambulating independently at the last follow-up, including 2 patients who had a disease duration exceeding 20 years.

In keeping with some reports showing weak to no significant correlation between size of *FGF14* expansion and age at onset or disease severity,^1,7,9^ we identified 3 patients who presented marked differences in age at onset and disease progression despite carrying an expansion of similar size (patient 2, 376 repeats; patient 3, 449 repeats; and patient 4, 424 repeats). Notably, patient 3 developed disease at age 28 years and had a more gradual disease progression (SDFS score of 1 after 23 years of disease duration) compared with patient 4 who developed disease at age 59 years and had an SDFS score of 3 after 9 years of disease duration.

One patient had evidence of mild axonal sensory polyneuropathy on electrophysiologic studies. Isolated vermis atrophy was observed in 4 patients (4/8; 50%) on brain MRI, while diffuse cerebellar atrophy was observed in 3 patients (3/8; 37%). Mild midbrain and pons atrophy was observed along with diffuse cortical atrophy in 1 patient. This patient had a comparatively more severe phenotype, with dystonia of the upper limbs, vestibular areflexia, and was wheelchair dependent. Although it is unknown whether these findings are pathologically related to GAA-*FGF14* ataxia, an extensive workup did not reveal an alternative diagnosis.

Of the GAA-*FGF14*–negative patients, we identified 4 patients who carried a non-GAA expansion (2 (GAAGGA)_n_, 1 (GAAGAAA)_n_, and 1 [(GAA)_4_(GCA)_1_]_n_). Non-GAA expansions in *FGF14* are likely not pathogenic, as previously shown in segregation studies.^1^

## Discussion

In this large and diverse Brazilian cohort, *FGF14* (GAA)_≥250_ expansions were present in 9% (8/93) of patients with unsolved adult-onset ataxia. We identified the first cases of GAA-*FGF14* ataxia in patients of admixed American ancestry and African descent. This observation supports the wide distribution of this novel GAA repeat expansion in populations of diverse ethnic backgrounds.

Our report expands the phenotypic spectrum of GAA-*FGF14* ataxia, with a patient of our cohort presenting before the age of 30 years. This result highlights the need to screen for *FGF14* expansions in patients with early-onset ataxia to fully define the age at onset spectrum of GAA-*FGF14* ataxia. The expansion size of our patient presenting at age 28 years was smaller compared with that of other patients in this cohort who developed disease later in life, which is in keeping with some previous reports showing weak or lack of correlation between age at onset and expansion size.^1,7,9^ This observation suggests that additional yet unknown factors may modify age at onset in GAA-*FGF14* ataxia.

Episodic symptoms and downbeat nystagmus have previously been shown to be common phenotypic features of GAA-*FGF14* ataxia in some, but not all, patient cohorts.^1,7,9^ In our cohort, only 1 patient had episodic symptoms while none had downbeat nystagmus. These results suggest that episodic features and downbeat nystagmus may not be recurrent in all patients. Nonetheless, the core phenotype of our patients, namely a late-onset slowly progressive cerebellar syndrome with frequent oculomotor signs, is consistent with previous reports.^1,2,7,9^

We also identified a patient carrying biallelic GAA repeat expansions, which has been previously reported in a few cases.^1,9,11^ This patient displayed a relatively rapid disease progression. Additional longitudinal follow-up is needed to fully chart disease progression and phenotypic spectrum in these patients. Furthermore, extrapyramidal signs were found in 2 patients (22%), raising the possibility that this feature may be part of the phenotypic profile of GAA-*FGF14* ataxia.

In conclusion, our study suggests that GAA-*FGF14* ataxia is a common genetic cause of unsolved adult-onset ataxia in the Brazilian population. Our findings also confirm the wide distribution of this novel GAA repeat expansion in populations of diverse ethnic backgrounds.
